# Contamination in complex healthcare trials: the falls in care homes (FinCH) study experience

**DOI:** 10.1186/s12874-020-00925-z

**Published:** 2020-02-27

**Authors:** K. Robinson, F. Allen, J. Darby, C. Fox, A. L. Gordon, J. C. Horne, P. Leighton, E. Sims, P. A. Logan

**Affiliations:** 1 Research and Innovation, Nottingham University Hospitals NHS Trust, Nottingham, NG7 2UH UK; 2 Division of Rehabilitation, Ageing and Wellbeing, Nottingham, UK; 3 Norwich Medical School, University of East Anglia, Norwich, UK; 4 Division of Medical Sciences and Graduate Entry Medicine, University of Nottingham, Derby, UK; 5 East Midlands Collaboration for Leadership in Applied Health Research and Care (EM-CLAHRC), Nottingham, UK; 6 University Hospitals of Derby and Burton NHS Foundation Trust, Derby, UK; 7 School of Medicine, University of Nottingham, Nottingham, UK; 8 Norwich Clinical Trials Unit, University of East Anglia, Nottingham, UK; 9 Nottingham CityCare Partnership NHS organisation, Nottingham, UK

**Keywords:** Falls, Contamination, Randomised controlled trials, Care home research

## Abstract

**Background:**

Trials are at risk of contamination bias which can occur when participants in the control group are inadvertently exposed to the intervention. This is a particular risk in rehabilitation studies where it is easy for trial interventions to be either intentionally or inadvertently adopted in control settings. The Falls in Care Homes (FinCH) trial is used in this paper as an example of a large randomised controlled trial of a complex intervention to explore the potential risks of contamination bias. We outline the FinCH trial design, present the potential risks from contamination bias, and the strategies used in the design of the trial to minimise or mitigate against this. The FinCH trial was a multi-centre randomised controlled trial, with embedded process evaluation, which evaluated whether systematic training in the use of the Guide to Action Tool for Care Homes reduced falls in care home residents.

Data were collected from a number of sources to explore contamination in the FinCH trial. Where specific procedures were adopted to reduce risk of, or mitigate against, contamination, this was recorded. Data were collected from study e-mails, meetings with clinicians, research assistant and clinician network communications, and an embedded process evaluation in six intervention care homes.

During the FinCH trial, there were six new falls prevention initiatives implemented outside the study which could have contaminated our intervention and findings. Methods used to minimise contamination were: cluster randomisation at the level of care home; engagement with the clinical community to highlight the risks of early adoption; establishing local collaborators in each site familiar with the local context; signing agreements with NHS falls specialists that they would maintain confidentiality regarding details of the intervention; opening additional research sites; and by raising awareness about the importance of contamination in research among participants.

**Conclusion:**

Complex rehabilitation trials are at risk of contamination bias. The potential for contamination bias in studies can be minimized by strengthening collaboration and dialogue with the clinical community. Researchers should recognise that clinicians may contaminate a study through lack of research expertise.

## Background

Randomised controlled trials (RCT) are considered one of the most reliable research methods for evaluating whether healthcare interventions are effective [1]. The use of randomisation to allocate participants to the intervention or control (comparator) ensures that the baseline characteristics of each group are as similar as possible [2]. If both groups are treated in exactly the same way, apart from the intervention they receive, then any observed differences can be attributed to the intervention. Randomised controlled designs work well if research protocols are expertly designed and adhered to but deviation from protocol can lead to introduction of bias. Randomised controlled trials are only ethically permissible where there is genuine uncertainty over whether the intervention is superior. As such, it is ethical to withhold the intervention from one group of participants.

Lewis and Warlow define bias as ‘*any departure from the truth’* [3] and as such the introduction of bias can influence the findings of a randomised controlled trial. There are different types of bias that can be introduced throughout the design and conduct of a randomised controlled trial. This discussion paper will focus on one form - *contamination bias*.

Contamination bias in a randomised controlled trial can be described as “*when members of the ‘control’ group inadvertently receive the treatment or are exposed to the intervention”* [4]*.* This may then minimise the difference in the observed outcomes between the control and intervention groups. Given the level of investment in randomised controlled trials to evaluate healthcare interventions it is important to consider how to manage the risks of contamination bias to ensure trials can produce reliable and a robust conclusion to develop clinical practice. Pragmatic trials where research is delivered in a real world setting are advantageous in providing evidence of whether an intervention actually works in routine clinical settings with wider generalisability and acceptability [5], however such trials have less control over trial conditions. The Cochrane group in a systematic review of interventions to prevent falls in care homes identified the need for researchers to consider the interaction of usual care with the intervention [6] which could introduce contamination bias and impact on trial outcomes. There is a concern that the neutral outcome of rehabilitation trials may be a result of control participants receiving a diluted version of the intervention.

Pragmatic trials of complex interventions are particularly at risk of contamination bias because they involve multiple components, multiple stakeholders and a range of organisations. This provides multiple opportunities for either the intervention or control to deviate from practices stipulated in the research manual. As a consequence, practices in the intervention and control arms can start to overlap.

The aim of this discussion paper is to provide an understanding of the possibilities for contamination bias within rehabilitation trials which involve a complex intervention, and to offer insights that might be valuable to researchers who are conducting such trials. We use the Falls in Care Homes (FinCH) trial as an example of a large randomised controlled trial of a complex intervention. We outline the FinCH trial design, present the potential risks from contamination bias, and the strategies used in the design of the trial to minimise or mitigate against this. Recommendations for clinicians and researchers are presented.

## The falls in care homes (FinCH) experience

### The falls in care homes (FinCH) trial

Falls are three times more frequent in care home residents than in community dwelling older people [7] and there is currently no conclusive evidence to guide the management of falls in care home residents [6]. The Guide to Action Tool for Care Homes is a multifactorial falls risk assessment and action process that has been co-designed by clinicians, care home staff, carers and researchers [8]. The assessment and tailored intervention process is consistent with NICE guidelines for managing falls in older people [9].

The FinCH trial was a multi-centre randomised controlled trial, with embedded process evaluation, evaluating whether systematic care staff training and implementation of the Guide to Action Tool for Care Homes reduced falls in care home residents. NHS falls specialists aimed to train 80% of care staff in those care homes randomly allocated to the intervention arm. The one-hour standardised training included raising awareness of the importance of managing falls as well as how to complete the Guide to Action Tool. After training care staff were advised to complete the Guide to Action tool with their residents to identify, assess and then take action to minimise the risk of falls.

Trial recruitment was completed in January 2018 with 87 care homes and 1698 care home residents recruited across 10 UK sites. Outcome data on the number of falls, equipment use, medication changes, activities of daily living, quality of life and primary care visits were collected at three monthly intervals up to 12 months after randomisation. Further information on the FinCH trial is available in the published protocol [10].

### Potential risks of contamination bias within FinCH

FinCH was a complex rehabilitation trial where the intervention and trial procedures involved interactions between clinicians, care homes, residents, researchers and wider stakeholders, such as commissioners and regulators of care [11]. These interactions could lead to a change in behaviour and potentially a change in usual care, even in control settings where exposure to the intervention was intended to be prohibited. The potential risks to introducing contamination bias are summarised in Table 1. It is acknowledged that the points raised in this discussion article outline potential sources of contamination bias and whether these sources actually change behaviours and which sources are more relevant remains unclear.

**Table 1:** Potential mechanisms for introducing contamination bias within the FinCH trial

Mechanism for potential risk	Potential action impacting on the trial
Detail of the intervention in the study documentation	FinCH trial study documentation outlined that the trial was evaluating the guide to action tool as a way of reducing falls for care homes residents. This documentation was discussed with care home managers to allow informed consent. Care home managers could review their existing falls management strategies and consider using the Guide to Action tool.
NHS clinicians delivering training package to care home staff	Falls specialists had access to the confidential training manual which formed the intervention. Clinicians could share this with other colleagues and care home staff considering this as best practice. Clinicians may have changed their behaviour and practice in their day to day clinical practice following their involvement in the FinCH trial. This might not be a conscious process.
Care home staff and managers move between homes	Care home staff that received the FinCH intervention training may move to a care home in the control arm and share their knowledge and skills. Conversely skills in the intervention homes could be lost following the training if care staff leave the home.
Publishing the development of the FinCH intervention and the feasibility trial	There has been a growing trend to publish trial protocols and development work to allow transparency in the research process [12]. The development of the Guide to Action tool was published by the research team and available to access online. The feasibility trial which established the trial procedures for the FinCH trial was also published and indicated a positive trend for the intervention.
Unable to blind therapists, care staff and care home residents to whether they are allocated to the intervention or control arm	Awareness of the group allocation could influence the response to subjective outcomes such as the quality of life measures completed by care home staff and residents.
Promotion of the study findings throughout the duration of the trial	Researchers are encouraged to engage with a wide range of stakeholders throughout the research process to maximise impact and to prepare for impact at the end of the study. The FinCH study was discussed in a wide variety of forums which included Enabling Research in Care Home (EnRICH) forums, TV news bulletins, and national conferences, care home communities of practice, commissioning groups and patient and public involvement groups.

As part of the FinCH study an embedded process evaluation was conducted in six care homes to explore delivery of the intervention, trial processes and the care home perspective. Data from the process evaluation provided an insight into the issues of contamination within the trial and quotes are presented below to highlight the potential mechanisms for contamination within FinCH. Clinicians who acted as trainers for the intervention were known to the homes due to their NHS clinical roles:

*“And then, when I’m in there, on my other roles, they’re giving me feedback on what they’ve done and how they’ve done it, which is really good as well, because we wouldn’t get that if we, I wasn’t working in them, so, it’s quite nice to know that they’re actually cracking on with the … the tool and using it, so, that’s quite nice.”* (NHS Falls specialist delivering the intervention)

This positive assessment does however highlight that those delivering the GtACH training (and advocating its use in this setting) had other roles and responsibilities which took them across the regional care home sector, including to work with care homes randomised not to receive GtACH. There was potential for practitioners, who believed in the benefit of the tool, to modify their practice in line with its recommendations even in control homes:

*“I think, I personally really like it. I think it’s a really good tool. I’m hoping we’ll, it will be around for a while and we can all use it, because it’s just got such a very good practical emphasis to it”* (NHS Falls specialist delivering the intervention)

As shown in Table 1, information promoting the research, describing the intervention, or other initiatives which might impact upon normal care, had a broad reach with potential to influence practice in the control arm. Dissemination events can therefore become a source of contamination when the new way of working is described to care home managers in order to encourage them to participate:

*“We went along to a seminar, open event and there was somebody representing the FiNCH study*” (Care Home Manager (A))

For larger care home chains this is further compounded by the way that companies decide which interventions to use in their homes and the communication that takes place between homes and their management teams. Corporate management could make decisions which modify practice in either the intervention or control arms.

*“We can’t just suddenly decide we’re going to use a different type of thing, but what we can do is recommend and put together and put forward ideas. And say, right, look, this has been really useful, we should add that to our information, or the information that you’ve got on that form is much better than what we’re doing, why are we not using that*?” (Care home manager (B))

High staff turnover and changes within management were highlighted as difficulties for training care staff in the intervention resulting in potential dilution of expertise in delivery of the intervention and the potential for staff to move to a care home within the control group and share their knowledge and skills of the intervention:

*“I think, they’re very busy places, and, but what I think, from my experience, is that a lot of staff leave you know, it’s quite fluid from a staffing point of view.”* (NHS Falls specialist delivering the intervention)

These data highlight that there are multiple risks of contamination within randomised control trials of complex interventions and strategies for reducing contamination are needed throughout the methodological design.

### Strategies used in the design of the FinCH trial

In the design and conduct of the FinCH trial a range of strategies were used to reduce the potential for contamination bias which are presented in Table 2.

**Table 2:** Strategies used to minimise contamination in FinCH

Mechanism for potential risk	Strategies used in FinCH to minimise the risk
Detail of the intervention in the study documentation	Discussion by research staff at the set-up meetings about the importance of continuing with usual care for the control group to act as a comparator.
NHS clinicians delivering training package to care home staff	Falls specialists were asked to sign a confidentiality agreement to state that they wouldn’t share the training manual.
Care home staff and managers move between homes	Data on the number of care home staff leaving and starting at each home was collected to allow discussion of the results in view of this data. It was however not feasible to collect data on where new staff were joining from and where staff leaving were going to work, however summarised numbers allowed a description of the frequency and extent of movement of care home staff.
Publishing the development of the FinCH intervention and the feasibility trial	The intervention manual was not published prior to the study completion.
Unable to blind therapists, care staff and care home residents to whether they are allocated to the intervention or control arm	Blinding of research assistants collecting the primary outcome of the number of falls. The FinCH administrator telephoned care homes prior to the research assistant visiting to remind the home that the RA should remain blinded to the group allocation. Cluster randomisation was used at the level of each care home to ensure that the training and delivery of the Guide to Action Tool was delivered across the whole home.
Promotion of the study findings throughout the duration of the trial	Meetings with clinical teams and commissioning groups to emphasise that the study findings were not yet ready to implement into practice. The content of the intervention was not described in detail when the study team were invited to present the on-going trial information and conferences and high profile impact events.
FinCH intervention developed using best available evidence and national guidelines	Dialogue with clinical teams who were preparing to deliver the FinCH intervention in control sites. The study team engaged in discussion with clinical teams and commissioners regarding the FinCH trial and the risk of delivering the FinCH intervention in control care homes during the trial. Strategies were put in place to allow the clinical teams to continue to deliver and develop their commissioned services in care homes not involved in the FinCH study. The study team ensured they were offering continued engagement with clinical teams reporting on the progress of the study, and directing them to current evidence that was likely to help them to develop strategies to implement their falls programmes, once the outcome of the FinCH trial was known.

### Management of issues throughout the trial

Throughout the trial the study team were made aware of six clinical initiatives being developed where systematic training delivered by healthcare professionals in falls prevention for care homes was planned. These initiatives included components of the FinCH intervention and were planned to be delivered in the FinCH study sites, potentially including homes in the control group. The FinCH intervention had multiple components and was developed in line with the best available evidence. National guidelines included elements of the FinCH intervention and, therefore as clinical teams develop their services in line with national guidelines, they were at risk of introducing contamination.

The strategy for minimising the risk of contamination was to openly engage with clinical teams and care homes in order to raise the potential issues of adapting the full intervention before formal evaluation. By being alert to emerging initiatives, the study team were able to engage in discussion with the clinical teams and commissioners to determine how to minimise the impact of the methodological quality of the trial. Clinical teams were encouraged and supported to deliver and develop their commissioned services in care homes not involved in the FinCH study. Continued engagement included reporting on the progress of the study as it ran, and directing clinical teams to current evidence that would help them implement their falls programmes outside the context of homes involved in FinCH.

## Discussion

Transparency in trials is increasingly important to demonstrate research integrity [12]. However, this transparency has the potential to create contamination risks [12], specifically in complex intervention rehabilitation trials where a network of people are involved in delivering the intervention [13]. In order to get a trial started and ensure recruitment targets are met, engagement with clinical services, health and social care professionals and commissioners are essential. This dialogue is needed to raise the profile of the trial and to attract interest in participation. While this engagement is important, increasing dialogue, publicity and networking with clinical teams and commissioners, who may or may not understand the complexities of conducting and achieving a trusted outcome of a definitive trial, increases the risk of contamination bias.

The FinCH experience demonstrates the potential for contamination by presenting studies at public events. However careful the study team were about revealing too much of the intervention detail, clinical teams that were persuaded by the idea of a systematic falls programme in care homes had an interest in implementing the intervention as soon as possible. The Chief Investigator of the trial and Principal Investigators in each of the study sites maintained close contact with their clinical networks. By doing so, the trial team got to hear about clinical services and commissioners who were planning on implementing a similar intervention, with similar resources, and were able to intervene. It is possible that research teams which were less clinically connected could have failed to recognise or been unable to respond to these challenges. The opportunity to talk with those commissioning and delivering health and social care, about implementing the intervention prior to the evidence that it was effective, minimised the potential risk of contamination bias.

We believe that the FinCH experience is not an isolated one when conducting clinical trials. The need to clearly describe usual care and consider how it can interact with the intervention has been highlighted by the Cochrane group in a systematic review of interventions to prevent falls in care homes [6]. This review identified poor quality evidence for multifactorial interventions drawing limited conclusions for practice. Further, high quality primary research was recommended which considered the methodological issues of the interaction of usual care with the intervention group.

Changes to usual care have been reported by clinicians in other complex rehabilitation trials such as the AVERT trial (A Very Early Rehabilitation Trial for Stroke) [14]. The qualitative process evaluation identified that, over the years that the trial was running, some clinicians considered that their routine care moved towards the early and intensive mobilisation which was being evaluated in the trial [15]. This study group have recognised the issue of contamination bias and are developing methods for monitoring contamination in complex rehabilitation trials [16].

The gradual change in practice over time which was seen in AVERT highlights a different aspect of the potential for contamination bias. In FinCH the study team had to mitigate against a more sudden change in practice with commissioners and clinical services wanting to immediately adapt their practice to include the FinCH intervention. A gradual shift and a sudden shift in practice both highlight the need for a nuanced, observant, responsive and adaptive approach to communications with research participants and the health and social care communities with which they interact.

It is acknowledged that the points raised in this discussion article outline potential sources of contamination bias and whether these sources actually change behaviours and which sources are more relevant remains unclear.

### Recommendations

The following recommendations are based on the learning from the FinCH experience.

- Identify and monitor potential bias in the conduct of randomised controlled trials of complex interventions
- Research teams should strengthen the dialogue with the clinical community to allow awareness of clinical developments that could impact on usual care and the clinical trial outcomes
- Increasing research capacity and capability within care home staff and social care.

## Conclusion

Pragmatic trials of complex interventions are particularly at risk of contamination bias because they involve multiple components, multiple stakeholders and a range of organisations. The potential risks and strategies to mitigate these risks need to be considered and monitored throughout the trial. The potential for contamination bias in studies can be minimized by strengthening collaboration and dialogue with the clinical community. Researchers should recognise that clinicians may contaminate a study through lack of research expertise.

## Data Availability

The datasets used and/or analysed during the current study are available from the corresponding author on reasonable request.
